# Things Worth Seeing in New-York

**Published:** 1863-06

**Authors:** 


### THINGS WORTH SEEING IN NEW-YORK.

Astor Library, free to all from 9 A.M. until sunset. Attendants will hand any book called for, to be used in the room. Lafayette Place, near Eighth Street, one block east of Broadway. 116,000 volumes.

Barnum's Museum, 222 Broadway, near Astor House. Twenty-five cents admission. Open from 8 A.M. until 10 P.M.

Bible House, on Fourth Avenue, one block east of Broadway, through Eighth Street, seven stories, occupying one whole block of ground, having cost $310,000. It employs three hundred persons, pays out four hundred and fifty thousand dollars a year, and in a year issued eight hundred and fifteen thousand Bibles and Testaments, in every variety of style and binding, from thirty cents for a complete Bible, up to twenty dollars each. The paper is received on the pavement, and is delivered in the seventh story a complete Bible.

Book-Making.—The most extensive printing-establishment in America is that of John A. Gray, Esq., on Frankfort Street, three blocks east of the City Hall, six stories, running twenty-six printing-presses, employing between two and three hundred men, women, boys and girls, within the building, and turning out every day an incredible amount of work, from a common pasteboard card up to bills, pamphlets, newspapers, magazines, and books in every style; and every thing well done, under the direction of one man, through that ceaseless vigilance, energy, firmness, and equanimity essential to all important positions; the pledge for even a temporary employment in the mammoth establishment being an engagement to be punctual, industrious, careful, quiet, clean, obedient, just and gentle in speech—qualities fit to be enumerated daily at the breakfast-table of every family in the land. Let them be "learned by heart" by every child that lives.

Central Park, reached by city cars, from Astor House, for five cents, by Third, Sixth, and Eighth Avenue lines; 844 acres; cost, to January 1, 1861, $7,600,000; appropriation for 1860, $2,500,000; total cost of purchase and improvements, up to January 1, 1861, $10,100,000. It is five miles from the Battery, is two and a half miles long, and half a mile broad; laid out by Frederick Law Olmsted, born in Conn., Lieutenant E. L. Viele, Engineer-in-Chief.

Cooper Institute, junction of Third and Fourth Avenues, built at an expense, including the ground, of over $630,000, by Peter Cooper, born in New-York City, Feb. 12, 1791. When completed, the noble man gave it to the city, to be devoted to the elevation of the working-classes of his birthplace, by instruction, without charge, in ordinary daily occupations, in sanitary, social, agricultural, and political science, and teaching addressed to the eye, the ear, and the imagination. The rents of the ground-floor are intended to pay all the expenses of keeping the building in perfect order. He was born poor, worked hard in a hatter's shop until he was seventeen, then learned coach-making. He built, at Baltimore, after his own design, the first locomotive engine ever used on this continent. Peter Cooper still lives. His name will be held in affectionate and respectful remembrance by millions yet unborn. Library and reading-room free to males and females.

Greenwood Cemetery is visited by great numbers. Most of the omnibuses convey you to South Ferry for six cents; ferriage, two cents; by Hamilton Avenue boats, from which horse-cars take you to the cemetery, five miles, for six cents. Carriages can be had at the gates, for one dollar an hour, for one or four persons. Intelligent drivers will point out the most striking monuments, with items of their history. Opened September 5, 1840, and up to Dec. 31, 1860, had received 81,325 of the dead.

Paintings, by the great masters, ancient and modern, from the twelfth century to the present time, at The Institute of Fine Arts, 625 Broadway. It includes the celebrated Dusseldorf Gallery, and the Jarves Collection, and is the largest and most *recherché* collection of paintings on this continent. Valuable additions are being constantly made. Admission, twenty-five cents.

Photographic Galleries are free to all, and will afford visitors the means of passing an hour with the highest satisfaction. The most prominent, in alphabetical order, are, Anson, Brady, Frederick, Gurney, Johnson, and Mead, all on Broadway.

Printing.—One of the greatest wonders of the city, and of the world, is the printing-press at *The World's* office, 37 Park Row, nearly opposite the Astor House. It can turn off twenty-five thousand impressions in an hour. It is made up of fourteen thousand seven hundred and thirty distinct pieces, weighs fifty thousand pounds, is fifteen feet broad, sixteen feet high, forty feet long, and cost thirty thousand dollars. Fifty years ago, it required two men nearly one hour to print a hundred newspapers. Any gentleman or lady, on application at the office, will have its working shown them.

